# Understanding the organisational culture of district health services: Mahalapye and Ngamiland health districts of Botswana

**DOI:** 10.4102/phcfm.v7i1.907

**Published:** 2015-11-30

**Authors:** Oathokwa Nkomazana, Robert Mash, Nthabiseng Phaladze

**Affiliations:** 1 Faculty of Medicine, University of Botswana, Botswana; 2 Division of Family Medicine and Primary Care, Stellenbosch University, South Africa; 3 School of Nursing, University of Botswana, South Africa

## Abstract

**Background:**

Botswana has a shortage of health care workers, especially in primary health care. Retention and high performance of employees are closely linked to job satisfaction and motivation, which are both highest where employees’ personal values and goals are realised.

**Aim:**

The aim of the study was to evaluate employees’ personal values, and the current and desired organisational culture of the district health services as experienced by the primary health care workers.

**Setting:**

The study was conducted in the Ngamiland and Mahalapye health districts.

**Method:**

This was a cross sectional survey. The participants were asked to select 10 values that best described their personal, current organisational and desired organisational values from a predetermined list.

**Results:**

Sixty and 67 health care workers completed the survey in Mahalapye and Ngamiland districts, respectively. The top 10 prevalent organisational values experienced in both districts were: teamwork, patient satisfaction, blame, confusion, job insecurity, not sharing information and manipulation. When all the current values were assessed, 32% (Mahalapye) and 36% (Ngamiland) selected by health care workers were potentially limiting organisational effectiveness. The organisational values desired by health care workers in both districts were: transparency, professional growth, staff recognition, shared decision-making, accountability, productivity, leadership development and teamwork.

**Conclusions:**

The experience of the primary health care workers in the two health districts were overwhelmingly negative, which is likely to contribute to low levels of motivation, job satisfaction, productivity and high attrition rates. There is therefore urgent need for organisational transformation with a focus on staff experience and leadership development.

## Abstrait

**Comprendre la culture organisationnelle des services de santé de district: les districts de santé de Mahalapye et Ngamiland au Botswana**.

**Contexte:**

Le Botswana a une pénurie de personnel de santé, surtout dans les soins de santé primaire. La fidélisation et la bonne performance des employés sont étroitement liées à la satisfaction et à la motivation au travail, qui sont le plus élevées quand les valeurs personnelles et les objectifs des employés sont atteints.

**Objectif:**

Le but de l’étude était d’évaluer les valeurs personnelles des employés, ainsi que la culture organisationnelle actuelle et voulue des services de santé de district vécue par le personnel de santé primaire.

**Lieu:**

L’étude a été menée dans les districts de santé de Ngamiland et Mahalapye.

**Méthode:**

C’était une étude transversale. On a demandé aux participants de choisir dix valeurs qui décrivent le mieux leurs valeurs organisationnelles personnelles actuelles et désirées à partir d'une liste prédéterminée.

**Résultats:**

Soixante des 67 membres du personnel de santé ont complété le sondage dans les districts de Mahalapye et Ngamiland, respectivement. Les dix valeurs organisationnelles les plus importantes dans les deux districts étaient : le travail d’équipe, la satisfaction des patients, les reproches, la confusion, l'insécurité professionnelle, la non communication des informations et la manipulation. Quand toutes les valeurs actuelles ont été évaluées, on a noté que 32% (Mahalapye) et 36% (Ngamiland) des valeurs sélectionnées par le personnel de santé pouvait limiter l'efficacité organisationnelle. Les valeurs organisationnelles désirées par le personnel de santé des deux districts étaient: la transparence, le développement professionnel, la reconnaissance du mérite des employés, le partage des prises de décision, la responsabilité, la productivité, le développement du leadership et du travail d’équipe.

**Conclusions:**

L'expérience des travailleurs de santé primaire dans les deux districts sanitaires était largement négative, ce qui probablement contribue aux faibles niveaux de motivation, de satisfaction professionnelle, de productivité et aux taux d'attrition élevés. Il est donc urgent de faire des transformations organisationnelles en mettant l'accent sur l'expérience du personnel et le développement du leadership.

## Introduction

Primary health care has been identified as an essential part of the health system when countries strive to provide universal access to quality cost effective health services.^1^ Effective primary health care services in many low-to-middle-income countries are, however, limited by high disease burdens, dysfunctional health systems and shortage of appropriately skilled and sufficiently motivated healthcare workers.^2,3,4,5,6^

Job satisfaction and motivation are strongly associated with retention and high performance of health care workers.^5,7,8^ Motivation, however, is complex as it is influenced by multiple intrinsic and extrinsic factors.^5,7,8,9^ Nevertheless, employees are known to have higher levels of productivity, commitment and creativity in organisations where their personal values and goals can be expressed or realised. These workers are said to have a kind of ‘psychological contract’ with the organisation.^10,11^ Successful organisations therefore will highly value their employee's satisfaction and experience in order to enhance their engagement, retention and the organisation's performance.^10,11,12^

Botswana's national health policy (2010) and the 10 year Integrated Health Services Plan (2010–2020) seek to ensure that there is ‘an appropriately skilled, motivated, well distributed and productive workforce for the provision of quality health services effectively and efficiently, to all the people living in Botswana.’^13,14^ The two policies subscribe to the following core values: ‘ethics, equity, ownership, evidence base, innovation, gender equity, client satisfaction, skilled staff retention and circulation and partnership’. The national Ministry of Health, which is the custodian and driver of policy and strategic planning, has the following core values: ‘customer focus (consistently meeting customer expectations), *botho* [providing service with respect, kindness in a friendly manner], timeliness (always delivering services on time to clients), equity (equal service delivery to all regardless of religion, social status or geographical location), teamwork (working together for a common goal) and accountability (responsible, liable and answerable for one's actions)’.^13^ All the district health management teams (DHMTs) espouse the values of the Ministry of Health. It is notable that the Ministry of Health, and hence the DHMTs, place no value on staff experience or satisfaction despite the plan to have a motivated and productive health workforce.

Shortage of health care workers and their inequitable distribution were identified as major bottlenecks to the delivery of quality health services in Botswana.^6,14^ The health workers, especially doctors, are also more concentrated in urban areas and hospital services compared to rural areas and primary care clinics.^2,6,14^ The government has used training (both in and outside the country), recruitment of expatriate workers as well as bilateral agreements with China and Cuba to address the shortage of health professionals in the public health system. Conversely, many Batswana (citizens of Botswana) health professionals trained in other countries have remained in the host countries on completion of their training.^2,6,14^ In addition, a significant number of those who returned, and a number of those trained locally have migrated from the public to the private or non-governmental sectors or have left the country.^6^ (Thupayagale-Tshweneagae G. Migration of Nurses in Botswana. Botswana Nursing Council. Gaborone 2009, personal communication).

Administratively, Botswana is divided into 28 health districts, and primary health care is provided through a network of 265 primary care clinics (101 with maternity beds), 343 health posts and 861 mobile clinic sites.^2^ The health facilities have been grouped into functional clusters centred around a larger clinic with maternity beds. Clinical services in the clusters are coordinated by cluster heads who are senior nursing officers. Management of all health services, including recruitment and deployment of health care workers (HCWs), procurement and distribution of equipment and drugs, is centralised at the national Ministry of Health headquarters. DHMTs were created in 2010 to administer healthcare services in their respective districts. Being a relatively recent structure many of the managers at the health districts are still being appointed and there is uncertainty about their roles.

Ineffective human resources and health care management systems, and inadequate supportive supervision that does not focus on improving staff satisfaction and experience have been identified as important determinants of low health worker motivation and retention in Botswana's public health service.^6^ This manuscript reports on the findings of a survey to evaluate the HCWs experience of the primary care organisation within Ngamiland and Mahalapye districts. The survey evaluated the organisational culture and was also intended to inform the work of a cooperative inquiry group that was initiated in the Ngamiland district. The last-mentioned group had been established to improve supportive supervision by the DHMT and cluster heads and to evaluate if this would increase staff motivation, experience and retention. The cooperative inquiry group was seen as the intervention in a quasi-experimental study and its findings will be presented later in separate articles.

## Methods

### Study design

A cross sectional survey of personal values, current organisational culture and desired future organisational culture, from the health workers’ perspective, was conducted in Ngamiland and Mahalapye health districts.

The values assessment was based on a conceptual framework that recognised the interrelation between the individual personality and character on the one hand and the organisational culture and structures or systems on the other (Table 1).^11^ At the individual level, one's internal values drive one's personality and are expressed through actions and behaviours as one's character. At the collective level the organisation's values drive its culture and are expressed in its collective actions, structures, procedures and processes.

**TABLE 1:** Four quadrants of human systems (Adapted from R Barrett).^12^

Variables	Individual	Collective
Objective (External)	Character: Individual actions and behaviours	Structures and procedures: Collective actions, behaviours and processes
Subjective (Internal)	Personality: Individual values and beliefs	Culture: Guiding values, attitudes that limit, shared strategic vision

Organisations therefore, like individuals, are living systems which possess internal values and external behaviours.^11,12^ The survey tools supplied by the Barrett's Value Centre make the internal individual and collective values visible and allow an assessment of the alignment between the four quadrants shown in Table 1.^11,12^ Highly functioning organisations have greater alignment or congruence between the four quadrants of this model. Personal alignment refers to the degree of congruence between one's individual values and behaviour, whilst structural alignment refers to the alignment between organisational values and structures or procedures. Values alignment refers to the extent to which individual values are congruent with organisational values and mission alignment refers to how well individual behaviour is congruent with organisational behaviour and procedures.^10^ A system that is well aligned across the quadrants will have employees that are more engaged and motivated, with high organisational performance.^12^ This model implies that a system or organisation that wants to improve its performance must work on alignment in and between all four quadrants simultaneously as they are interdependent.^11^ At the organisational level, the leadership has the greatest influence over creating and maintaining the prevailing culture and therefore transformation almost always involved leadership transformation.^15^

In the model developed by Richard Barrett the values selected in the survey can also be assigned to seven different levels of organisational consciousness (Table 2). The seven levels of consciousness were originally developed from Maslow's hierarchy of needs, and the distribution of values provides insight into what the organisation currently needs from its employees and what the employees need the organisation to focus on in the future. A well-functioning organisation will have positive values across all the levels.^11,12,15^ In the bottom three levels there can be both limiting as well as positive values. Limiting values are values which are usually detrimental to the functioning of the organisation and limit the organisation's performance. In the model the total percentage of all values chosen that are potentially limiting values corresponds to the organisational entropy, which is a measure of the amount of dysfunction and wasted energy in the system.^12,15^

**TABLE 2:** Seven levels of organisational consciousness (adapted from R Barrett).^12^

Consciousness level	Example of collective values
7. Service to humanity and social contribution	Social responsibility, long-term perspective, future generations, ethics, compassion, humility
6. Making a difference to the local community or health district	Environmental awareness, community involvement, strategic partnerships, employee fulfilment, coaching, mentoring and leadership development
5. Sense of purpose and strong internal cohesion	Shared vision and values, commitment, creativity, enthusiasm, integrity, generosity, fairness, honesty, openness, transparency and trust
4. On-going improvement and employee participation	Adaptability, accountability, empowerment, teamwork, goals orientation and continuous improvement
3. High performance and quality of care	Reliability, quality, efficiency, productivity, excellence, best practice, systems and processes. Bureaucracy†, hierarchy†, confusion†, arrogance†, hoarding information†and complacency†
2. Relationship with colleagues and patients	Loyalty, open communication, patient experience and friendship. Blame†, internal competition†, rivalry†, favouritism†andmanipulation†
1. Survival: Resources and safety	Sufficient budget, equipment, employee health and safety. Control†, greed†, chaos†, caution†, job insecurity†, exploitation†and micromanagement†

†, Limiting values.

### Setting

The research was performed in the health districts of Ngamiland (population: 96 356) and Mahalapye (population: 117 492), which are serviced by 29 and 40 primary care facilities (clinics and health posts) respectively. According to the 2011 National Health Policy, 80% of the Botswana population use public health services.^13^

The DHMT is led by a head, who is usually a doctor with public health training, with heads of preventive, curative and corporate services reporting to him or her, whilst he or she reports to the Deputy permanent secretary, clinical services. The clinic clusters are managed by cluster heads who are nursing officers based at the larger clinics. Each clinic in turn is managed by a nursing sister-in-charge, who reports to the cluster heads. The majority of the HCWs employed at these clinics and health posts are nurses and midwives with small numbers of pharmacy technicians, social workers, health education assistants and medical officers (generalist doctors). The doctors are based at the larger clinics and support the smaller facilities through regular outreach.

### Study population

All the primary HCWs in Ngamiland and Mahalapye health districts were eligible to participate in the survey, whereas support staff, such as drivers and cleaners and HCWs based in hospitals, were excluded. The research teams visited most primary care facilities to deliver the survey forms and for those facilities that were not visited the forms were given to the cluster heads. The plan was to recruit all consenting health workers on duty in the facilities during the two weeks of data collection. All the HCWs on duty during the two weeks of data collection were invited to complete the survey. Cluster heads were also invited to complete the survey as they are also based in the primary health care facilities.

### Data collection

The questionnaire was a standardised and validated tool developed by the Barrett Value Centre.^16^ The participants were asked to select 10 values, from a predetermined list, that best described their personal values, the organisational culture as they currently experienced it and the culture they desired in the district health service. The researchers also collected demographic data which included, gender, age, name of health facility, length of stay at the facility and job title. The anonymous questionnaire was administered in English, which is the normal language of communication in the organisation. The tool was piloted for understanding amongst HCWs working at the University of Botswana clinic who were considered to be of the same level of training and experience as the study population. The hardcopy survey was self-administered by the participants, and trained research assistants provided clarification as required. The collated questionnaires were then captured in the password controlled Barrett Value Centre website by one of the research assistants.

### Data analysis

The Barrett's Value Centre analysed the data and provided the study team with the results and a report. The results were descriptive and provided the numbers and frequencies for the selected values. The results were presented in figures and words and mapped to the seven levels of organisational consciousness.

The results were presented to the HCWs in each district in order to explore the meanings of the selected values. They were also presented to the Ngamiland District Health Management Team and cluster heads as part of the input into the cooperative inquiry process.

## Ethical consideration

Approval to conduct the study was given by the University of Botswana Institutional Review Board, the Ngamiland District Ethics Committee, the Stellenbosch University Health Research Ethics Committee (Protocol Number: S13/03/051) and the Botswana Ministry of Health, Health Research Development Committee (Reference No: PPME 13/18/1 V11 (368)). Written consent was obtained from each study participant.

## Results

### Demographical data

In Mahalapye, 60 primary HCWs on duty during the data collection period completed the survey compared to 67 in Ngamiland (Table 3). All those on duty during the data collection period agreed to participate in the survey, although one form from Mahalapye and five from Ngamiland were incomplete and therefore excluded from the data analysis. The health workers were recruited from 25 of Mahalapye's 40 (63%) clinics and health posts and 19 of Ngamiland's 29 (66%) primary care facilities. The majority of participants were female in Ngamiland (64%) and Mahalapye (66%).

**TABLE 3:** Age, length of service in the district and the different staff categories.

Variables	Mahalapye *N*=60 *n*(%)	Maun *N*=67 *n*(%)
**Staff categories**		*n*(%)
Doctors	1 (1.7)	1 (1.7)
Nurses	55 (91.7)	60 (89.5)
Others	4 (6.8)	3 (4.5)
Unknown	0	3 (4.5)
Mean age (range) years	35.7 (23–51)	35.4 (23–60)
Mean length of service in district (range) years	5.6 (1–20)	4.9 (1–21)

Most of the HCWs in primary care in these districts were nurses (Table 3). The staff category labelled ‘others’ included social workers, pharmacists and health education assistants. Health education assistants are specifically trained for 18 months to be a link between primary care facilities and the community.

### Ngamiland health district values

Table 4 shows the top 10 values in rank order for the Ngamiland respondents’ personal values, current experience of the organisation and desired future experience of the organisation. The values in italics are personal values that were also desired organisational values and those in bold were current organisational values that health workers wanted to continue to experience in the future. None of the personal values were currently experienced in the organisation.

**TABLE 4:** Top 10 personal, current organisational and desired future organisational values for Ngamiland health district.

Personal values	*N*= 62 n	Current organisational values	*N*= 62 n	Desired organisational values	*N*= 62 n
Accountability§	40	Working in isolation†	22	Transparency	26
Caring	30	Confusion†	21	Leadership development	20
Trust	24	Job Insecurity†	20	Teamwork‡	20
Respect	23	Information sharing	20	Staff recognition	17
Honesty	22	Working strictly by the rules†	19	Accountability§	16
Cooperation	20	Blame†	18	Productivity	16
Commitment	19	Not sharing information†	18	Shared decision making	15
Openness	18	Teamwork‡	18	Professional growth	14
Compassion	16	Patient satisfaction	17	Staff engagement	13
Responsibility	16	Manipulation†	16	Staff health	12

†, limiting value;

‡, values that are both current organisational values and desired organisational values;

§§§, values that a both personal values and desired organisational values.

Seven of the top 10 current organisational values were potentially limiting (marked with L) and only three were positive (Table 4). When all the limiting values were analysed there was a cultural entropy rate of 36%, which is a high level of entropy ‘requiring cultural and structural transformation, and leadership development’.^10^

There were no limiting values amongst the top 10 desired organisational values. These include only one currently experienced organisational value, teamwork, and one personal value, accountability (Table 4).

### Mahalapye health district values

Table 5 shows the top 10 values in rank order for the Mahalapye respondents’ personal values, current organisational and desired future organisational values. The values in italics were personal values that were also desired organisational values and those in bold were current organisational values that health workers wanted to continue to experience in the future. None of the personal values were currently experienced in the organisation.

**TABLE 5:** Top 10 personal, current organisational and desired organisational values: Mahalapye health district.

Personal values	*N*= 59 n	Current organisational values	*N*= 59 n	Desired organisational values	*N*= 59 n
Accountability§	37	Teamwork‡	22	Transparency	24
Caring	24	Blame†	18	Professional growth	18
Respect	20	Community involvement	17	Staff recognition	18
Commitment	19	Confusion†	17	Shared decision making	15
Compassion	19	Job insecurity†	15	Accountability§	14
Honesty	19	Patient satisfaction	15	Productivity	14
Cooperation	17	Not sharing information†	14	Organisational growth	12
Responsibility	17	Long hours†	13	Staff fulfilment	12
Trust	15	Control†	12	Empowerment	11
Fairness§	14	Manipulation†	12	Fairness§	11
		Professionalism	12	Leadership development	11
		Shared vision	12	Mission focus	11
		Working strictly by the rules†	12	Teamwork‡	11

†, limiting value;

‡, values that are both current and desired organisational values;

§, values that a both personal values and desired organisational values.

Eight of the current organisational values were potentially limiting (marked with L) and five were positive. When all the limiting values are analysed there was a high cultural entropy rate of 32%.

None of the top 10 desired organisational values were potentially limiting. Teamwork was the only desired value currently experienced in the organisation. Two of the top 10 personal values, accountability and fairness, were desired for the future organisation (Table 5).

### Comparison of Mahalapye and Ngamiland health district values

Primary HCWs in Mahalapye and Ngamiland share nine common personal values (Table 6) and two dissimilar values, namely, fairness (Mahalapye) and openness (Ngamiland).

**TABLE 6:** The top 10 common personal current and future organisational values for Mahalapye and Ngamiland.

Personal values	Current organisational values	Desired organisational values
Accountability	Teamwork	Transparency‡
Caring	Patient satisfaction	Professional growth‡
Respect	Blame†	Staff recognition‡
Commitment	Confusion†	Shared decision-making‡
Compassion	Job insecurity†	Accountability‡
Honesty	Not sharing information†	Productivity‡
Cooperation	Manipulation†	Leadership development‡
Responsibility	Working strictly by the rules†	Teamwork
Trust	-	-

†, Limiting values; ‡, Desired values that are not part of the prevalent organisational culture.

Eight of the top 10 values in the current culture were also shared by the two districts. These included two positive values and six limiting values, as shown in Table 6. Working in isolation was another potentially limiting value currently experienced in Ngamiland whilst long hours and control were experienced in Mahalapye. Furthermore, Mahalapye had the following positive values: community involvement, shared vision and professionalism compared with information sharing for Ngamiland. None of the personal values of the health workers were experienced in either district.

The primary HCWs in Ngamiland and Mahalapye also shared 8 of the top 10 desired organisational values (Table 6). These included seven new (bold) values and one that is currently experienced in both districts. Moreover, Mahalapye health workers also selected the following desired values: organisational growth, staff fulfilment, empowerment, fairness and mission focus, whilst those in Ngamiland chose staff health and staff engagement.

**FIGURE 1:**
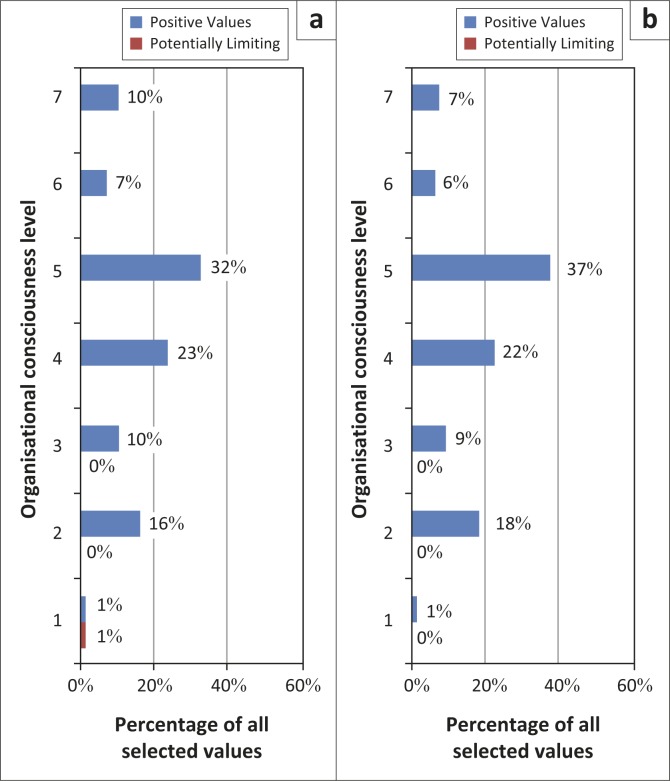
(a) Personal values of Mahalapye HCWs mapped to organisational consciousness level. (b) Personal values of Ngamiland HCWs mapped to organisational consciousness level.

Figure 1 shows the percentages of all personal values of HCWs in Mahalapye and Ngamiland according to the level of organisational consciousness. HCWs in the two districts had almost identical distributions of personal values that were concentrated at Level 5 followed by Levels 4 and 2.

**FIGURE 2:**
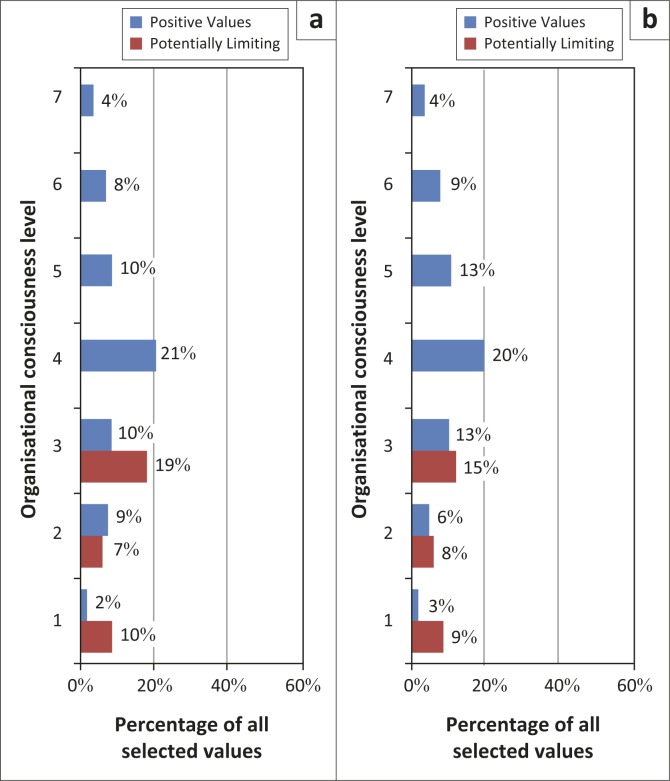
(a) Current organisational values Mahalapye health districts mapped to organisational consciousness level. (b) Current organisational values Ngamiland health districts mapped to organisational consciousness level.

The distribution of current organisational values for the two districts was concentrated at Level 3 (Figure 2). The employees’ experience of the organisations at this level was largely negative. The organisations were experienced as bureaucratic and people felt confused, isolated and needed more information. This was followed by Level 4 with an organisational emphasis on teamwork and Level 2 with a positive focus on relationships with patients, but a negative experience of relationships in the organisation due to a sense of being both blamed and manipulated. At Level 1 there was also a concern about job security.

**FIGURE 3:**
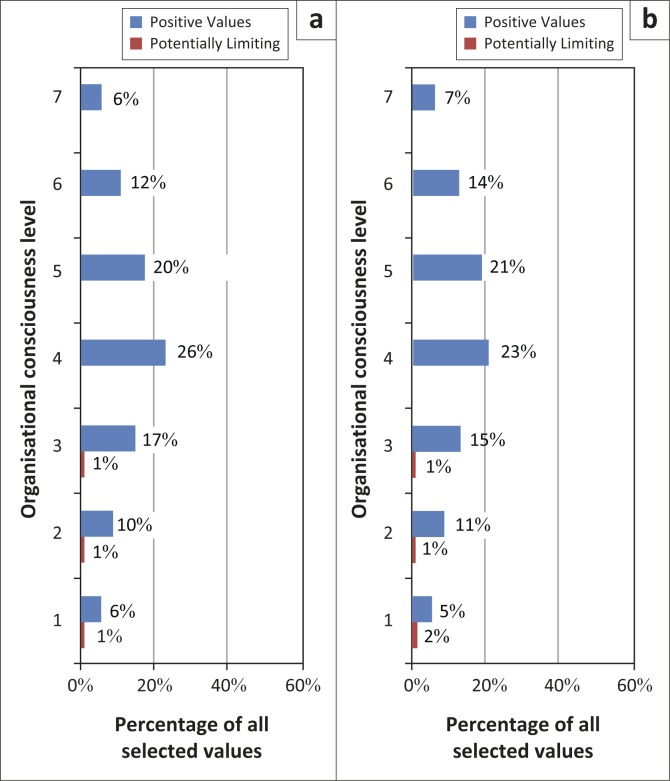
(a) Desired organisational values for Mahalapye health districts mapped to organisational consciousness level. (b) Desired organisational values for Ngamiland health districts mapped to organisational consciousness level.

The distribution of the desired values for the two districts was similarly concentrated at Level 4 with a need for more accountability and shared decision-making, followed by Level 5 with a call for more transparency to build alignment and internal cohesion (Figure 3). Level 3 represented a positive commitment to improved productivity and professional growth. The other levels suggested a need for more attention to staff welfare and more recognition of the work they were doing as well as a call for development of the leadership within the organisation.

Table 7 summarises the feedback from the health workers in both districts on their interpretation of the values seen in the current organisational culture. ‘The current organisational values reflect the employees’ perceptions of what their organisation focuses on and how it behaves. These attributes provide a picture of the working environment, the positive aspects of the business, and its potential problem areas. These are a description of what the situation is between these groups at this point in time’.^11,12^

**TABLE 7:** Implications of the top current organisational values for Mahalapye and Ngamiland health districts as interpreted by healthcare workers.

Value	Implication
Teamwork	People in the two districts were aware that they could accomplish more together than alone. The continued appeal for teamwork in the future was a call to build a complete team. The people believed the team was incomplete and as such ineffective as some critical health care cadres were either missing or in short supply. The people also wanted their contribution to the team effort to be recognised and rewarded (*staff recognition*).
Blame	Some people in both organisations were being mistreated by being held accountable for the misdeeds of others. Poor communication in a bureaucratic environment had led to employees also blaming management for problems and vice versa. The call for *accountability*implied that employees wanted to be acknowledged and rewarded for good performance and made to account for poor performance. They also placed a very high value on *transparency*and *honesty*to facilitate accountability.
Community involvement	The clinics are embedded in the community and in Mahalapye the respondents saw the district health services as engaged in local issues and working closely with the community. The interaction with the community though was not always perceived as positive by health workers, who often felt unfairly criticised by the community for things they had no control over. Unrealistic expectations and interference by some community and political leaders was also disruptive to efficient running of the clinics.
Confusion	The DHMTs were a fairly recent invention whose implementation was not properly communicated to the health workers, who were still unsure of the district health system governance structure. Moreover the people in both districts experienced a lack of clarity as well as mixed messages from the national Ministry of Health and DHMT, which left them unsure of motives and directives. This confusion was compounded by poor communication. The request for *leadership development*implied they saw the need for learning opportunities that would help their leaders in uniting and guiding the employee population. The people were also calling for clear transparent communication and staff engagement to bring about common understanding, especially in issues that affected the way they work.
Job insecurity	There was fear and uncertainty in both districts about the people's ability to keep their jobs. As such, people tended not to question the status quo, but worked strictly by the rules. An environment of blame, poor information sharing, control and manipulation all heightened this insecurity. Some human resources management policies were construed as unclear and unfairly applied. There was a call for *professional growth*as employees sought learning and career opportunities in their organisations. The people also sought transparency and fairness in implementation of human resources policies.
Patient satisfaction	Each of the districts emphasised the fact that they existed to meet the needs of their patients.
Not sharing information	The people in the two districts did not have access to the truths that would provide clarity and understanding about what was going on in the organisation. Information flow was often just from top to bottom and not vice versa. Even the information from the top often did not trickle down to all the health facilities and especially not to all employees. These employees called for *transparency*and *openness*and that those giving directives should actively provide clarity around their motives and orders in order to promote shared understanding.
Long hours	In Mahalapye people were overworked. They acknowledged that some clinics had few patients, but these sought health care around the clock leaving very little time for nurses working alone in the clinic to rest. Increased numbers of programmes without increase in staff had also stretched this limited resource with minimal reward and recognition. They were therefore burnt out and demoralised. Employees were therefore calling for an organisational focus on *staff fulfilment*and *staff recognition*.
Control	In Mahalapye the people were not empowered to do their work, but were rather micromanaged. The employees sought to be empowered to utilise and develop their skills and be held accountable for their actions. They also wanted to share in decision making. Leadership development at all levels was also wanted to create a more open and supportive management style.
Manipulation	Nurses in both districts felt exploited as they were expected to do tasks traditionally done by doctors, pharmacists and laboratory technicians without any extra reward. The clear call for *shared decision making*, *staff recognition*and *staff fulfilment* was intended to address this.
Professionalism	Mahalapye had a focus on high standards of care and conduct.
Shared vision	Mahalapye health district also recognised the importance of a sense of purpose and direction that united and inspired their efforts.
Working strictly by the rules	In both districts bureaucracy and the status quo hampered the employees efforts who tended to keep quiet and adhere to the business norms of the health districts. The call for *empowerment*(Mahalapye) and *staff engagement*(Ngamiland), *professional* and *leadership development*were made in order to free the innovation and creativity of the staff.
Information sharing	Those from Ngamiland were focused on exchanging knowledge and resources across the business in order to help empower employees’ efforts. Despite this, a similar number of respondents experience lack of information sharing as the predominant culture.
Working in isolation	Ngamiland is a large remote and rural district where many health workers work alone. These health workers felt detached from the rest and unsupported. Consequently, depression and loneliness were experienced by many. The employees, therefore, sought support for their basic well-being (*staff health*). They also want to be involved in decisions about their deployment as well as opportunities (*staff engagement*) to fully utilise their talents and skills.

## Discussion

This study has investigated the personal values of the HCWs in the Mahalapye and Ngamiland health districts as well as the perceived current and desired organisational culture of the health districts. Organisational culture has been strongly linked to employee satisfaction and organisational commitment and hence their retention.^17^ An understanding of the prevailing organisational culture in the Botswana district health system and how this is likely to affect retention of primary HCWs is critical to guide organisational transformation.

The results of this study showed that the HCWs’ personal values were focused on demonstrating ownership for their actions, being truthful and trustworthy. They also placed emphasis on fostering kind and supportive interactions with the people around them. The prevailing culture in the districts, on the other hand, was predominantly perceived as potentially limiting and likely to cause frustration amongst employees and hinder organisational performance. None of the personal values of the HCWs were currently experienced in either district where a culture of blame, control, manipulation, poor communication and working strictly by the rules dominated. HCWs in both districts additionally experienced high levels of job insecurity. It is also notable that more than a third of the current organisational values were perceived as constraining organisational performance. The incongruence between personal and organisational values is likely to negatively affect job satisfaction, performance and organisational commitment.^12,17^

The personal values of the primary HCWs in the two health districts in Botswana were almost identical to their counterparts in the Cape Town primary care services where the primary health care services were also equally experienced as negative with levels of entropy above 30%.^18^ Similar organisational dysfunction has also been reported by Kenyan HCWs in Kenya's district hospitals.^19^

With the exception of teamwork and customer focus (patient satisfaction), the current organisational values were not aligned to the espoused values of the national Ministry of Health and district health services of *botho* [kindness, compassion, respect], integrity, timeliness and accountability.^14^ These espoused values, however, are similar to the actual personal values of the employees, implying that the staff have the potential to manifest the espoused values of the Ministry if given a chance to do so. Interestingly, the Western Cape government embraces similar core values of caring, competence, accountability, integrity, responsiveness and respect, despite its mostly negative employee experiences.^20^ This incongruity between personal and organisational values as well as espoused and actual values is very likely to result in low health worker motivation, high attrition and poor patient outcome.^8,10,11,12^

Primary care workers in the two districts desire organisational transformation that concentrates on building a sense of shared purpose, internal community and employee participation. They were in agreement that a more supportive and innovative culture with a focus on transparency, professional growth, staff recognition, shared decision-making, accountability, productivity and leadership development should replace the current largely limiting culture. The HCWs also wanted to see teamwork continue as part of the future culture of the district health services. A more supportive and innovative culture together with considerate supportive supervisors has been associated with increased job satisfaction and organisational commitment.^17^ Organisational culture and leadership styles determine the work environment, which can be either helpful or detrimental to organisational effectiveness and job satisfaction.^21^ Furthermore, the perception of a limiting work environment and paucity of supportive supervision is strongly correlated with low job satisfaction and intention to migrate.^22^ Alignment of the distribution of the desired organisational culture and personal values denotes that the health workers are capable of affecting the change they want to see.^18^

Botswana's human resources for health are heavily skewed towards cities and hospital care, displaying an ‘inverse care law,’^23,24^ despite the national Ministry of Health and the district health systems espoused value of equity.^14^ Attraction and retention of motivated and committed health workers to primary health care services, especially in rural areas, should therefore be prioritised. This mandates a new emphasis on employee experience with a focus on professional development, shared decision-making and staff engagement in the district health system.

The response rates were relatively low, but the study captured the views of all the different health care cadres working in two thirds of the health facilities of each district. The study was conducted in two of the 27 health districts in the country, which means the findings may not be applicable to the rest of the health system. The concordance in the personal and perceived organisational values in these two dissimilar districts, however, suggests that similar findings may be elicited in other health districts. The very strong similarity and distribution of the values and culture with Cape Town primary care services also suggests that this state of affairs may be prevalent in the region.

## Conclusion

The primary HCWs in Mahalapye and Ngamiland health districts perceive the organisational culture of the health districts to be mostly limiting organisational effectiveness. The health workers personal attributes were strongly correlated with the transformed culture they desired to see in their health districts, reflecting personal capacity to effect the changes. The employees’ personal attributes and those they want to see in the future transformed organisation are in keeping with what is known about organisational cultures and leadership styles that create employee satisfaction, organisational commitment and retention. Transformation of the leadership from a bureaucratic controlling style focused on keeping the status quo to a more supportive type is urgent in order to restore employee trust and retain them in primary care.

There is an urgent need for organisational transformation of the health care services at the district and national levels to translate the espoused organisational values to the felt culture. This transformation should accentuate employee experience with an emphasis on accountability, transparency, professional growth, staff recognition and shared decision making. Opportunities for professional growth as well as employee recognition are well known to improve health care worker motivation and retention.

Leadership have the greatest influence in creating and sustaining the prevailing organisational culture. Leadership development is therefore imperative at all levels of the health system for organisational transformation to succeed.

Implementation research to explore whether developing more supportive leaders will improve employee motivation and retention is required. Additionally, investigation of aspects of organisational transformation that will significantly impact staff motivation and retention in the Botswana primary health care services will need to accompany the transformation.
